# The Impact of Intermittent Fasting on Metabolic and Hormonal Profile in Patients with Polycystic Ovary Syndrome: A Systematic Review and Meta-Analysis

**DOI:** 10.3390/metabo15100654

**Published:** 2025-10-02

**Authors:** Iman Aolymat, Suhad Abumweis, Hafez Al-Momani, Diala Walid Abu-Hassan, Majd M. Albarakat, Ahmad Alzoubi, Mohammed Abu saleh, Ayah Khleaf Oleimat, Shaimaa Nasr Amin, Walaa Bayoumie El Gazzar, Ahmed Salem, Amin N. Olaimat, Heba A. Ali, Abd Al-Rahman Al-Shudiefat

**Affiliations:** 1 Department of Anatomy, Physiology and Biochemistry, Faculty of Medicine, The Hashemite University, P.O. Box 330127, Zarqa 13133, Jordan; ahmadalzoubi.md01@gmail.com (A.A.); mohammad.abusaleh025@gmail.com (M.A.s.); ayakhlaifolimat2003@gmail.com (A.K.O.); shaimaa@hu.edu.jo (S.N.A.); wallagazzar@hu.edu.jo (W.B.E.G.); asalem@hu.edu.jo (A.S.); 2 Department of Clinical Nutrition and Dietetics, Faculty of Applied Medical Sciences, The Hashemite University, P.O. Box 330127, Zarqa 13133, Jordan; suhadsuhad.abumweis@aau.ac.ae (S.A.); aminolaimat@hu.edu.jo (A.N.O.); 3 Department of Microbiology, Pathology and Forensic Medicine, Faculty of Medicine, The Hashemite University, P.O. Box 330127, Zarqa 13133, Jordan; hafez@hu.edu.jo; 4 Department of Physiology and Biochemistry, School of Medicine, The University of Jordan, Amman 11942, Jordan; d.abuhassan@ju.edu.jo; 5 Faculty of Medicine, Jordan University of Science and Technology, P.O. Box 3030, Irbid 22110, Jordan; mmalbarakat20@med.just.edu.jo; 6 Department of Physiology, Faculty of Medicine, Cairo University, Cairo 11562, Egypt; 7 Department of Medical Biochemistry and Molecular Biology, Faculty of Medicine, Benha University, Benha 13518, Egypt; 8 Division of Cancer Sciences, University of Manchester, Manchester M13 9PL, UK; 9 Department of Basic Dental Sciences, Faculty of Dentistry, The Hashemite University, P.O. Box 330127, Zarqa 13133, Jordan; heba_ali@hu.edu.jo; 10 Department of Medical Laboratory Sciences, Faculty of Applied Medical Sciences, The Hashemite University, P.O. Box 330127, Zarqa 13133, Jordan; abdsalem29@hu.edu.jo

**Keywords:** polycystic ovary syndrome, hyperandrogenism, insulin resistance, reproductive hormones, intermittent fasting

## Abstract

**Background:** Polycystic ovarian syndrome (PCOS) is one of the most prevalent reproductive, endocrine, and metabolic disorders inflicting women of childbearing age. Dietary interventions have gained interest as non-pharmacological approach to control obesity and metabolic disturbances. However, the effects of intermittent fasting (IF) on metabolic and hormonal profiles of PCOS patients is debatable. **Objectives:** We performed this systematic review and meta-analysis to explore IF’s effect on PCOS women’s metabolic and hormonal profile (PROSPERO: CRD42024511520). Eligible studies included IF interventions in women with PCOS, with metabolic and hormonal profiles being reported. **Methods**: A systematic literature search using three databases, including PubMed, SCOPUS, and Web of Science, was conducted. The systematic review was performed following PRISMA guidelines. **Results:** A total of four studies were included (*N* = 4). IF is not associated with significant change in BMI (MD = −0.200, 95% CI [−0.807, 0.407], *p* = 0.518). The analysis revealed that IF had no statistically significant impact on FBG (MD = −0.569, 95% CI [−9.955, 8.818], *p* = 0.906), HOMA-IR (MD = −0.862, 95% CI [−1.737, 0.014], *p* = 0.054), and FINS (MD = −2.749, 95% CI [−6.441, 0.943], *p* = 0.145). No significant change in TG (MD = −3.120, 95% CI [−9.624, 3.385], *p* = 0.347), total cholesterol (MD = −0.918, 95% CI [−2.960, 1.124], *p* = 0.378), and LDL levels (MD = −0.433, 95% CI [−1.224, 0.359], *p* = 0.284) between IF and pre-fasting or non-intervention diet groups. However, the explanation is limited by the small number of studies, duration of fasting regimes, and/or variations in fasting strategies. Sex hormone data were collected but were insufficient for a pooled analysis. **Conclusions:** Overall, our study suggests that IF is not an effective intervention to enhance BMI, glycaemic control, and lipid metabolism in PCOS patients. Nevertheless, the current conclusion is inconclusive and preliminary, as additional well-designed studies are required to support this conclusion.

## 1. Introduction

Polycystic ovary syndrome (PCOS) is one of the most frequent endocrinopathies among women in their reproductive age. The disorder approximately affects 20% of females around the world [1]. PCOS has a complicated etiology that involves a combination of genetic and environmental factors. The occurrence of PCOS can be caused by malfunction in various components of the hypothalamic–pituitary–ovarian and hypothalamic–pituitary–adrenal axes [2]. This heterogenous complex disorder is characterized by hyperandrogenism [3], obesity, and insulin resistance (IR) [4,5]. Women with PCOS are at increased risk of developing several comorbid metabolic, reproductive, and psychological disorders. Metabolic comorbidities include obesity, type 2 diabetes mellitus (T2DM), cardiovascular disorders, and dyslipidaemia [6]. Reproductive comorbidities include cancers of the endometrium and pregnancy-related complications, such as abortions, preterm labour, gestational diabetes, and preeclampsia [7]. Moreover, women diagnosed with PCOS are at higher risk of developing psychiatric disorders such as depression, anxiety, bipolar disorder, and obsessive–compulsive disorder (OCD) [8]. Therefore, providing early intervention and therapeutic measures to PCOS women who are at risk can prevent such potential health disorders.

Treatment options for PCOS include both pharmacological and non-pharmacological modalities, including exercise and diet [9]. Recently, several dietary interventions have been employed and proven effective in treating PCOS [10,11]. Intermittent fasting (IF), defined as alternating periods of fasting and feeding, has gained attention for its potential metabolic benefits, including weight loss, improved insulin sensitivity, and hormonal regulation. Emerging evidence suggests that IF is potentially relevant for women with PCOS, given the central role of metabolic disturbances in PCOS. Recent studies have investigated the role of different IF regimens in PCOS management [12,13]; however, the number of studies conducted in this context is limited [14], widely variable in design, fasting duration, and outcome measures. A contradictory body of literature has been published on IF’s effect on the metabolic and hormonal profiles of PCOS patients, with some studies suggesting that it may improve those profiles, whereas others suggest that IF has no apparent impact on improving metabolic and hormonal functions of PCOS patients. Although the positive impact of IF on the metabolic and hormonal profiles of PCOS patients has been documented in a small number of human and animal studies, the use of IF to treat PCOS is insufficiently supported. These gap in knowledge highlight the importance of systematic synthesis of the evidence. In order to develop evidence-based PCOS management plans, health care providers may find it helpful to perform additional research on the impact of IF on the metabolic and hormonal parameters in PCOS patients. Therefore, this meta-analysis aimed to evaluate the effectiveness of IF intervention in improving the metabolic and hormonal profiles in PCOS women. The current meta-analysis investigated the actual efficacy of IF on anthropometric measurement, glucose homeostasis, lipid profile, and reproductive hormones in PCOS patients.

## 2. Materials and Methods

### 2.1. Protocol Registration

This meta-analysis was organized and carried out in accordance with the Preferred Reporting Items for Systematic Reviews and Meta-Analyses (PRISMA) 2020 statement [15]. The study protocol was registered in the International Prospective Register of Systematic Reviews (PROSPERO) under the registration number CRD42024511520.

### 2.2. Data Sources and Search Strategy

A systematic literature search of human studies in three databases—PubMed, Scopus, and Web of Science—was conducted and completed on 12 February 2024 to identify studies investigated the impact of IF on metabolic and hormonal outcomes in women with PCOS. A compilation of keywords related to PCOS and IF was used to conduct the literature search. The details of exact search terms used in each database are reproduced in Appendix A exactly as implemented. These included combinations of Medical Subject Headings (MeSH) and free-text terms related to “intermittent fasting”, “time-restricted feeding”, and “polycystic ovary syndrome”, along with variations in spelling and phrasing adapted to the requirements of each database. Additionally, a manual search of the references list of included articles and relevant reviews was carried out to identify potentially eligible studies.

### 2.3. Eligibility Criteria

The following PICOS criteria were employed to include population (P): Females with PCOS; intervention (I): Intermittent fasting (IF); control (C): Regular diet vs. fasting or pre-fasting data vs. post-fasting data have been compared if the control group was not available; outcome (O): Body mass index (BMI), fasting blood glucose (FBG), fasting insulin (FINS), Homeostatic Model Assessment of Insulin Resistance (HOMA-IR), lipid profile (total cholesterol (TC), low-density lipoprotein cholesterol (LDL-C), and triglycerides (TG)), sex hormones (LH, FSH, and total testosterone (TT)), sex hormone-binding globulin (SHBG), and free androgen index (FAI); and study design (S): Retrospective cohort, cross-sectional, quasi-experimental studies, and randomized control trials (RCTs).

#### 2.3.1. Inclusion Criteria

Any intervention studies evaluating IF protocols in human females with PCOS, including retrospective cohort, cross-sectional, quasi-experimental studies, and RCTs.

#### 2.3.2. Exclusion Criteria

- Reviews, case reports, and case series studies.
- The article’s language is not English.
- No appropriate control.
- Animal studies.

### 2.4. Study Selection

Two independent reviewers, AZ and MA, were involved in the selection of the studies after the exclusion of duplicates using “Covidence” software (https://www.covidence.org/). Initially, the reviewers individually screened titles and abstracts of search results using “Rayyan” online software (https://www.rayyan.ai/). Then, full-text screening was conducted using previously identified eligibility criteria. A third reviewer (IA) was involved in resolving any disputes.

### 2.5. Data Extraction

The process of data extraction was conducted by two independent reviewers, AZ and MA. From each eligible study, the following study characteristics were extracted: the first author’s surname, publication year, study design, sample size, type of intervention and control, and intervention duration. Information regarding the study population, including age, BMI, and health status, was also extracted. From each eligible study, data on mean and SD for all outcomes of interest, including BMI, glucose homeostasis (FBG, FINS, and HOMA-IR), lipid profile (TC, LDL-C, and TG), hormones (LH, FSH, LH:FSH ratio, and TT), SHBG), and FAI. IA was involved in cross-checking data extracted by AZ and MA. SA was also involved in data double-checking for any computed values.

### 2.6. Quality and Risk-of-Bias Assessment

The risk of bias within individual studies included in this meta-analysis was evaluated using the Risk of Bias In Non-randomized Studies of Interventions (ROBINS-I) tool [16]. ROBINS-I assesses bias arising from confounding, selection of participants into the study, classification of interventions, deviations from intended interventions, missing data, measurement of outcomes, and selection of reported results.

Each included study was independently assessed by two reviewers (MA and SA) for these domains, with any discrepancies resolved through discussion when necessary. The risk of bias for each study was categorized as low, moderate, serious, or critical according to the guidelines provided by ROBINS-I.

### 2.7. Statistical Analysis

Comprehensive Meta-Analysis V2 (Biostat, Englewood, NJ, USA) was used to calculate the pooled effect size as mean difference (MD) and 95% confidence interval (95% CI) for continuous outcomes from the reported means and SDs of the post- and pre-treatment values in the individualized studies using a random-effect model, as we assumed that the true effect size varies across the trials. We used the mean difference to provide a clinically meaningful interpretation especially since we carried out the analysis on outcomes with well-established standardized measuring methods. Only data from pre–post treatment studies were pooled together to minimize heterogeneity. For some outcomes, the mean and its variance were estimated from their corresponding median and the first and third quartiles based on a method reported in the Cochrane Handbook (available from https://training.cochrane.org/handbook/current (accessed on 15 September 2025). In other cases, we calculated the within-individual correlation between the post- and pre-treatment periods and used it for the calculations of the pooled effect size.

Heterogeneity or variation between study results was tested using a standard chi-squared (χ^2^) test and I^2^, which describes the percentage of the variability in effect estimates attributed to heterogeneity rather than chance [17]. A guide to I^2^ interpretation is as follows: 0% to 40%: might not be important; 30% to 60%: may represent moderate heterogeneity; 50% to 90%: may represent substantial heterogeneity; and 75% to 100%: considerable heterogeneity. Unfortunately, subgroup analysis was not applicable due to the limited number of trials. Sensitivity analysis was performed for each of the analyzed outcomes.

## 3. Results

### 3.1. Search Results and Study Selection

**Figure 1:**
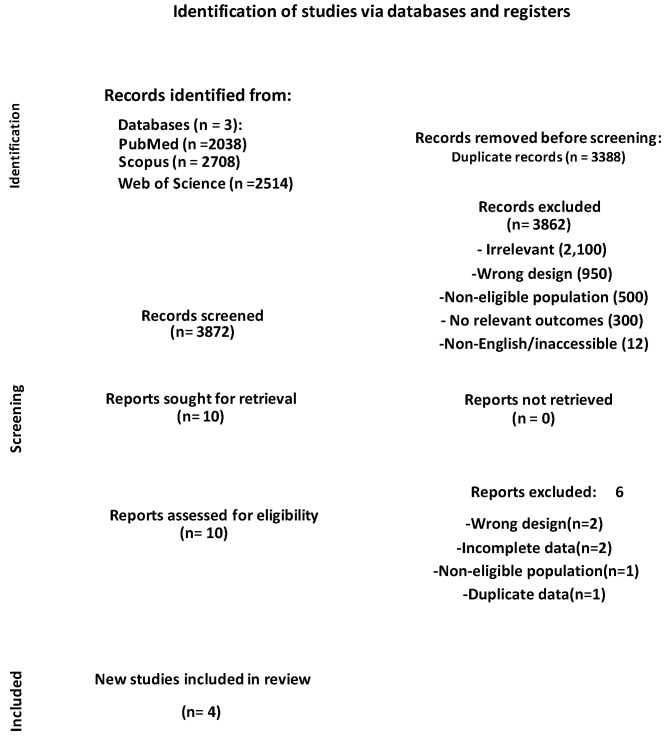
Preferred Reporting Items for Systematic Reviews and Meta-Analyses (PRISMA) diagram for the screening and selection of studies in this systematic review.

Our search through the three databases produced 7260 results. After removing 3388 duplicates, 3872 items were left for the title and abstract screening. During title and abstract screening, 3862 articles were excluded. The detailed list of exclusion criteria is presented in Supplementary Materials (Results S1). Then, ten articles were retrieved for full-text examination. Of these ten articles, six items were excluded due to reasons such as wrong study design (*n* = 2), insufficient or incomplete outcome data (*n* = 2), non-eligible population (*n* = 1), or duplicate data from an already included study (*n* = 1). At the end, four items were included in the meta-analysis. Figure 1 shows the PRISMA flow diagram recording the search and selection process.

### 3.2. Characteristics of Included Studies

Table 1 shows the characteristics of the four included studies in this meta-analysis. In general, the four studies evaluated the impact of IF on different parameters of PCOS. Feyzioglu et al. [18] implemented a six-week trial of eight-hour TRF, observing changes in anthropometrics, hormones, lipids, and fecal calprotectin in PCOS women [18]. Li et al. focused on a six-week trial of TRF in PCOS patients, reporting changes in weight, body fat, menstrual cycle regularity, and markers of hormonal and inflammatory imbalance [13]. Asemi et al. explored the impact of Ramadan fasting over four weeks on nitric oxide and glutathione, glucose, lipids levels, and overall antioxidant capacity in PCOS women [19]. Zangeneh et al. assessed the effects of Ramadan fasting on stress and key sex hormone levels in Iranian women with PCOS [20]. Li et al. [13] and Feyzioglu et al. [18] implemented structured, health care-supervised protocols with regular follow-ups and dietary monitoring. Asemi et al. [19] and Zangeneh et al. [20] conducted studies during Ramadan, where fasting was religiously observed but still involved clinical supervision for data collection and biochemical measurement.

### 3.3. Risk-of-Bias Assessment

**Figure 2:**
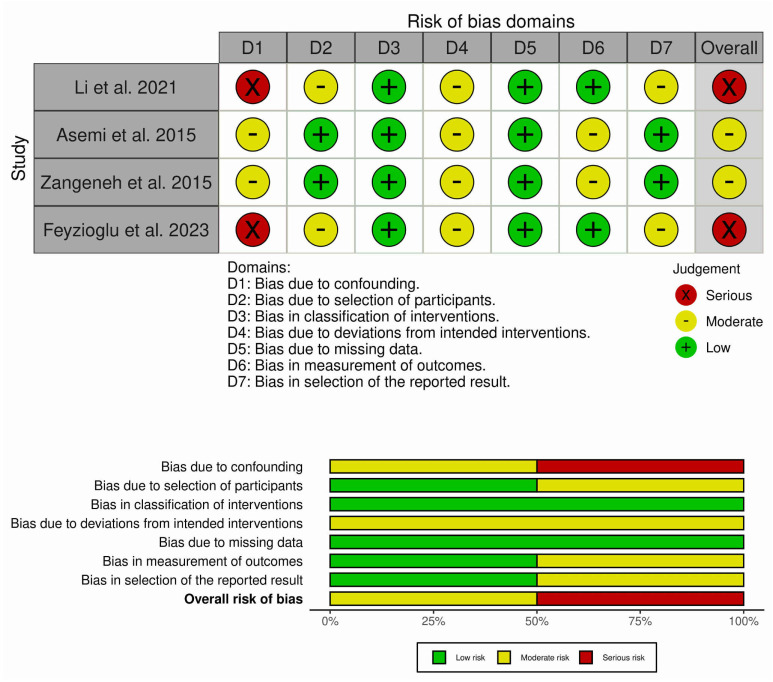
Risk of bias evaluation in the included studies [13,18,19,20] using the ROBINS-I tool. Each row represents a study, and each column corresponds to one of the seven bias domains. The last column indicates the overall risk of bias judgement. Colours represent the level of bias risk: green = low risk, yellow = moderate risk, and red = serious risk.

Figure 2 presents the domain-level risk of bias for each included study, using the ROBINS-I tool. By displaying results across all seven domains—confounding, participant selection, classification of interventions, deviations from intended interventions, missing data, outcome measurement, and selective reporting—the figure provides overview of methodological limitations. Asemi et al. [19] and Zangeneh et al. [20] were characterized by a moderate overall risk of bias, whereas Li et al. [13] and Feyzioglu et al. [18] demonstrated a serious overall risk of bias. Despite these concerns, outcome measurement methods were generally reliable, and missing data were minimal across studies.

Asemi et al.’s [19] study was assessed as having a moderate risk of bias overall. Although the investigators applied clear diagnostic criteria for PCOS and described the Ramadan fasting intervention well, the absence of a control group introduced a moderate risk of confounding, since changes in outcomes could have been influenced by factors unrelated to fasting. While all participants completed the study, reducing concerns about missing data, there was potential for deviations from the intended intervention because dietary and lifestyle behaviours were not strictly controlled. Outcome measurements such as insulin resistance and lipid profiles were collected using objective laboratory methods, but variability in timing and diurnal effects may have introduced some bias. Reporting appeared transparent, but given the design limitations, the study cannot fully isolate the effects of fasting from other influences.

Zangeneh et al.’s [20] clinical trial, comparing fasting and non-fasting PCOS patients, was considered to have a moderate risk of bias. The inclusion of a control group reduced, but did not eliminate, the risk of confounding, since lifestyle and circadian disruptions associated with Ramadan could have influenced results independently of fasting itself. Participant selection was well defined and based on accepted diagnostic criteria, and adherence to Ramadan fasting was objectively described; however, variability in the number of days fasted and altered sleep patterns may have introduced deviations from the intended intervention. Laboratory assays for cortisol, catecholamines, and sex hormones were reliable, but the small sample size increased the likelihood of random error. Reporting appeared complete, yet the limited scope of measured outcomes and potential unmeasured confounders led us to assign a moderate overall risk.

The single-arm pre–post intervention trial conducted by Li et al. [13] was rated as having a serious risk of bias, largely due to confounding. Without a concurrent control group, it is not possible to rule out that the observed improvements were due to natural variation or external influences rather than the time-restricted feeding intervention itself. Participant recruitment was relatively narrow (anovulatory PCOS women), limiting generalizability and raising some concerns about selection bias. While the intervention protocol was well defined and outcomes measured using validated biochemical and anthropometric methods, adherence relied on self-reporting. Missing data were minimal, as most participants completed the follow-up. Reporting was generally thorough but did not provide much information on adverse events or durability of effects. These factors together support the judgement of serious overall risk of bias.

Feyzioglu et al.’s [18] study carries a serious risk of bias. The lack of a control group meant that confounding factors could not be adequately addressed, particularly given the wide range of exclusion criteria applied during participant selection. Although the eight-hour time-restricted feeding protocol was well described, adherence was self-reported, which introduces uncertainty about how closely participants followed the diet. Missing data were minimized since reasons for exclusions were documented, and most outcomes were measured with reliable laboratory methods. However, selective reporting was a concern, as longer-term outcomes such as menstrual regulation were not fully presented. Taken together, these issues explain why this study was classified as having a serious risk of bias.

### 3.4. Effect of IF on Body Mass Index

**Figure 3:**
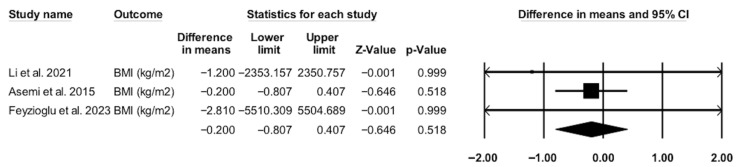
Forest plot of the body mass index (BMI) of intermittent fasting (IF) vs. pre-fasting or non-intervention regular diet. The x-axis represents difference in mean, with 0 indicating no effect. Each square represents an individual study, with the size proportional to its weight in the analysis; horizontal lines indicate 95% confidence intervals (CI). The diamond represents the pooled effect size, with its width corresponding to the 95% confidence interval [13,18,19].

The pooled analysis revealed no statistically significant difference in BMI between the IF group and the control or pre-fasting group among patients with PCOS (MD = −0.200 kg/m^2^, 95% CI [−0.807, 0.407], *p* = 0.518) (Figure 3). Only three studies contributed to this analysis. The wide confidence intervals suggest considerable uncertainty. It is worth to mention here that the small number of studies decreases both the strength of evidence and visual plot impact. The pooled analysis showed no statistically significant differences, which may reflect differences in IF protocols, short duration of study, measured outcomes, and differences in study participant characteristics such as BMI and PCOS phenotypes. Therefore, we cautiously interpret this finding and highlight the importance of larger, well-designed trails to support this observation.

### 3.5. Effect IF on Glucose Homeostasis (Fasting Glucose, Fasting Insulin, and Homeostatic Model Assessment for Insulin Resistance)

**Figure 4:**
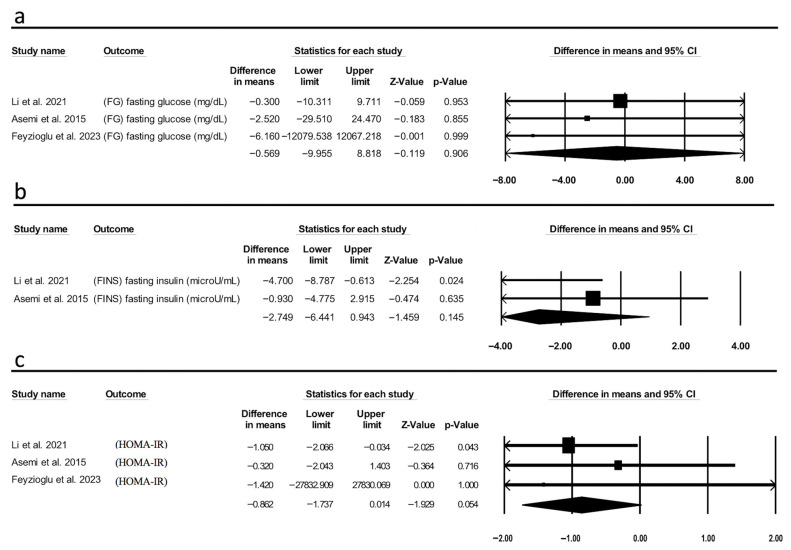
Forest plot of (**a**) fasting glucose (FG), (**b**) fasting insulin (FINS), and (**c**) homeostatic model assessment for insulin resistance (HOMA-IR) of intermittent fasting (IF) vs. pre-fast. The x-axis represents difference in mean, with 0 indicating no effect. Each square represents an individual study, with the size proportional to its weight in the analysis; horizontal lines indicate 95% confidence intervals (CI). The diamond represents the pooled effect size, with its width corresponding to the 95% confidence interval [13,18,19].

The pooled analysis revealed that IF had null impact on FBG between the two groups (MD = −0.569 mg/dL, 95% CI [−9.955, 8.818], *p* = 0.906, I^2^ = 0.00) (Figure 4a). Similarly, there was no significant effect observed on HOMA-IR (MD = −0.862, 95% CI [−1.737, 0.014], *p* = 0.054, I^2^ = 0.00) (Figure 4b). Additionally, a pooled analysis investigating the effect of IF on FINS yielded non-significant results (MD = −2.749 μU/mL, 95% CI [−6.441, 0.943], *p* = 0.145, I^2^ = 42.3) (Figure 4c). The number of included studies in this analysis was three, two, and three, respectively, which showed substantial variability in their results, suggesting weaker confidence in the estimates. Although the forest plot shows an important visual presentation, it is important to emphasize that the evidence is still low and should be considered exploratory rather than confirmatory.

### 3.6. Effect of IF on Lipid Profile (Triglycerides, Total Cholesterol, and Low-Density Lipoprotein)

**Figure 5:**
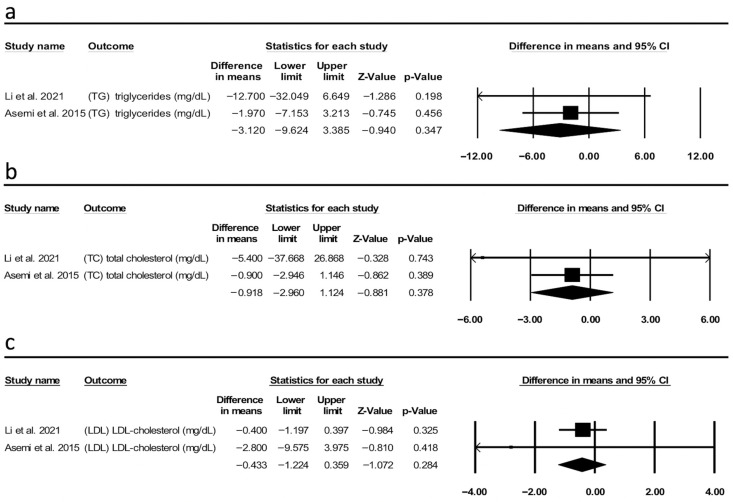
Forest plot of lipid profile (**a**) triglycerides (TG), (**b**) total cholesterol (TC), and (**c**) low-density lipoprotein-cholesterol (LDL-c) of intermittent fasting (IF) vs. pre-fasting or non-intervention regular diet. The x-axis represents difference in mean, with 0 indicating no effect. Each square represents an individual study, with the size proportional to its weight in the analysis; horizontal lines indicate 95% confidence intervals (CI). The diamond represents the pooled effect size, with its width corresponding to the 95% confidence interval [13,19].

The investigation into the effect of IF on lipid profile revealed no significant impact on TG, TC, and LDL levels (MD = −3.120 mg/dL, 95% CI [−9.624, 3.385], *p* = 0.347, I2 = 9.3 (Figure 5a); MD = −0.918 mg/dL, 95% CI [−2.960, 1.124], *p* = 0.378, I^2^ = 0.00 (Figure 5b); and MD = −0.433 mg/dL, 95% CI [−1.224, 0.359], *p* = 0.284, I^2^ = 0.00 (Figure 5c), respectively). The number of included studies in this analysis was only two studies. The evidence is limited and inconsistent, suggesting weaker confidence in the pooled estimates. It is important to emphasize that the results should be explained with caution. Moreover, this represents an important evidence gap: insufficient high-quality studies are present to draw solid conclusions about the impact of IF on lipid profiles in patients with PCOS.

### 3.7. Effect of IF on Sex Hormones

This study aimed to explore the effect of IF on the level of sex hormones, including LH, FSH, the LH:FSH ratio, TT, and SHBG in PCOS patients. Among the included studies, three out of four have investigated the changes in the reproductive hormones following IF. However, the included studies have provided insufficient information to calculate a pooled effect estimate. Therefore, in this section, we will summarize the key findings of the individual studies in terms of IF consequences on sex hormones. Zangeneh et al. studied the effect of Ramadan fasting on the levels of reproductive hormones in PCOS women compared to the non-fasting group. The study has shown that, following Ramadan fasting, blood levels of FSH, LH, and testosterone did not significantly change between the two groups [20]. The other study conducted by Li et al. [13]. evaluated the 8 h TRE impact on the reproductive hormones in women with PCOS. The study reported a significant enhancement in SHBG associated with a significant reduction in TT and FAI after the IF regimen. On the other hand, no significant change in serum levels in LH and FSH, and LH/FSH was observed after IF [13]. The last study demonstrated that after fasting, the levels of AMH, FSH, LH, TT, free testosterone, and FAI were significantly decreased when compared to the pre-fasting levels. Whereas the after-fasting level of SHBG has significantly improved compared to the pre-fasting level. Table 2 summarizes the individual study results for sex hormones. The included studies showed different perspectives in the effects of IF on sex hormones in women with PCOS, which may be attributed to variations in fasting protocols, duration, and hormonal assessment methods. Zangeneh et al. [20] stated that there are no significant changes in FSH, LH, or testosterone after Ramadan fasting, whereas Li et al. [13] and Feyzioglu et al. [18] reported significant improvements in hyperandrogenaemia markers—specifically reductions in TT and FAI and increases in SHBG—following TRF interventions. These different outcomes may be caused by differences in fasting duration (daytime fasting vs. TRF), dietary composition, and participant characteristics such as BMI and baseline hormonal profiles. Moreover, the variability in measured hormones (e.g., some studies reporting AMH, DHEAS, or free testosterone while others did not) underscores the need for standardized hormonal measures and consistent reporting in future studies.

### 3.8. Sensitivity Analysis

Sensitivity analysis revealed that the mean difference remained insignificant for BMI, FBG, TC, LDL, and TG. However, for the outcomes HOMA-IR (*n* = 3) and FINS (*n* = 2), removing one study resulted in significant findings, with values of MD = −1.050, 95% CI [−2.66, −0.034] and MD = −4.7 μU/mL, 95% CI [−18.787, −0.613], respectively.

## 4. Discussion

Our analysis included four independent studies that have evaluated the effect of IF on metabolic and hormonal outcomes of PCOS patients. The results showed that the IF intervention was not associated with a significant effect on metabolic and hormonal outcomes of patients with PCOS. Additionally, the heterogenous evidence and lack of comprehensive analysis of PCOS hormonal profile make it hard to draw a conclusion in this regard. These findings are potentially due to the small number of included studies, short fasting regimens, and variability in fasting strategies. As all changes observed are non-significant and are small in magnitude, they are likely not clinically relevant. Although heterogeneity statistics observed between studies are considered not important, differences in study design, particularly IF protocols, may have hindered the detection of significant differences in the outcomes included in this analysis.

In general, previous studies in adults without PCOS have suggested that IF is associated with a significant reduction in BMI. For example, a previous meta-analysis of six studies conducted by Harris et al. showed that IF was effective in the treatment of overweight and obesity in adults [21]. Another meta-analysis has shown that IF was associated with a significant reduction in BMI of patients with metabolic syndrome [22]. These comparisons should be explained cautiously because metabolic disturbances in PCOS is different from those in the general population and may modulate the effect of IF. Similarly, PCOS rat models have been shown to significantly lose weight following the IF intervention [12]. However, the findings of the present meta-analysis contradict the findings of those previous studies. According to Asemi et al.’s study, IF in PCOS patients was not associated with significant improvement in BMI [19]. This contradiction is potentially due to the differences in the fasting duration and regimen. Only four to six weeks of intervention have been included in the current meta-analysis, and the intervention regimens included 8 h fasting in a study, 16 h fasting in another, and Ramadan fasting in the remaining two studies, whereas the IF duration reported in the previous studies [21,22] has ranged from 1 to 26 weeks.

The available literature concerning how the IF could modulate glucose homeostasis and lipid profile is plenty but inconsistent; however, no previous meta-analysis has been conducted to examine the role of IF on the glycaemic control and lipid profile of PCOS patients. Several previous meta-analyses have established the favourable impact of IF on enhancing glucose homeostasis and lipid profile compared to other dietary interventions or no intervention. A recently published meta-analysis carried out by Zaman et al. examined the effect of TRF on cardiometabolic health. The study suggested that IF has reduced the levels of blood glucose, insulin, and TGs. By contrast, the IF intervention was not associated with a significant change in HOMA-IR, TC, LDL-c, and HDL-c [23]. Another meta-analysis of RCTs has shown that while IF improved insulin sensitivity (HOMA-IR), fasting insulin, cholesterol, and TG concentrations, it had no effect on FBG, LDL, and HDL levels [24]. Our meta-analysis elucidated that the IF in PCOS patients had no favourable effect on glucose and lipid metabolism. The data discrepancy among different studies is potentially attributed to variations in the research populations, demographic features, study designs, various patterns of fasting, and the heterogenous control groups. This highlights the importance of conducting more research in the future to reconcile conflicting results and gain a comprehensive understanding of the role of IF in the management of PCOS.

The recently published article by Vale-Fernandes et al. [25] can contextualize and interpret the findings of our study. Their research elucidated that PCOS and excessive body weight affect reproductive outcomes independently and synergistically. Interestingly, obesity was shown to independently worsen insulin resistance, hyperandrogenism, and metabolic disturbances. These obesity-mediated disturbances could make PCOS-associated endocrine disorders even worse. Moreover, the study has shown that women with PCOS exhibited higher levels of AMH, elevated LH: FSH ratio, and reduced FF progesterone levels, confirming these as signatures of PCOS regardless of BMI, and obesity mainly exacerbates metabolic dysfunction rather than modulating reproductive outcomes. In light of our findings, this interplay between obesity and PCOS has important implications. Heterogeneity or null results observed across IF interventions can be partially explained by obesity contribution to the disorder as women with PCOS who are obese/overweight showed different metabolic and hormonal profiles compared with lean controls. For example, the study has reported that IF ability to restore women’s androgen level or enhance insulin sensitivity was reduced by elevated BMI. Therefore, future studies should separate women with PCOS by BMI to determine whether IF regimens vary between groups. This could also help identify targeted groups which most likely respond to IF.

PCOS is characterized by imbalances in sex hormones, including elevated levels of androgens, LH, and AMH, and reduced levels of FSH [3]. One of the aims of this study was to systemically evaluate the impact of IF patterns on the reproductive hormones in PCOS patients. However, due to insufficient data reporting, we were unable to analyze this outcome of interest. It is important to acknowledge that this area of interest has not been extensively explored previously, and the available studies are small in number and short in duration. This highlights the need for standardized reporting of sex hormones, including the type of biological sample, the type of essay used, the specific time of sample collection, and the unit of measurement. Therefore, conducting further studies in the future to improve the available knowledge regarding the impact of IF on PCOS patients’ sex hormones is important.

Some limitations must be acknowledged when discussing the findings of this analysis. Firstly, the small number of included studies (*n* = 4) diminishes the strength of our conclusions. With this small number of included studies, formal assessment of publication bias is considered not feasible or meaningful. Therefore, this limitation is acknowledged as it decreases the confidence in the robustness of this study findings and emphasizes on the idea that future studies may alter the current conclusions. As a result, the findings of this study should be interpreted cautiously until more studies are conducted and evaluated. The observed null effect of IF on metabolic and hormonal profile of PCOS might be attributed to the short duration of included studies, which might be insufficient to detect a meaningful effect of IF. This highlights the requirement of longer-duration studies in the future. Other confounders such as age, BMI, and adherence to fasting regimens may have affected the outcomes of this analysis. Uncontrolled or inconsistent reporting of such confounders may have led to the variability in results. These variables should be considered in future studies. Moreover, the heterogeneity of IF protocols applied in the included studies, ranging from 8 h or 16 h fasting periods to religious fasting regimens is another limitation that might make it harder for comparisons and potentially lead to variations in outcomes. This necessitates the need for standardized interventions in the upcoming research. Additionally, RCTs are the gold standard studies for evidence-based medicine since they are established to reduce the risk of bias. It is worth mentioning here that the scarcity of RCTs in this area and the lack of RCTs in our meta-analysis, which makes the study design less robust, necessitates the demand for conducting RCTs in the future to establish the role of IF in the management of PCOS and to identify the optimal IF programme and duration to treat this condition.

In summary, our analysis showed null effect of IF on metabolic and hormonal profiles in PCOS. These findings reflect the limited number of studies, short study durations, variability in fasting interventions, and unexplored sex hormones. For clinicians and patients, IF cannot be recommended as a therapeutic intervention for PCOS at the present time. However, it may still provide a potential lifestyle approach that may be promising, taking in consideration its metabolic advantages in the non-PCOS population. More importantly, sex hormones, which are key to PCOS pathology, are still not explored extensively, showing a major evidence gap to investigate as indicated in this analysis. To improve the available knowledge, future studies with an appropriate design, a larger population, standard IF strategies, a longer duration, and comprehensive hormonal analysis are recommended.

## 5. Conclusions

Based on only four studies with heterogeneity, the findings of this systematic review and meta-analysis suggest that the role of IF in PCOS is inconclusive and preliminary. Statistically insignificant effects of IF were observed for metabolic and hormonal profiles, and the evidence base is limited to support clinical recommendations. Hormonal dysregulation lies at the core of PCOS pathology. However, a scarce number of studies have evaluated hormonal outcomes after IF intervention in PCOS. As a result, our meta-analysis highlights a significant evidence gap, namely, the lack of high-quality data on the role of IF in hormonal outcomes of PCOS patients. The results of this study should be explained cautiously.

Future research on the role of IF in controlling PCOS should prioritize larger, high-quality RCTs with standardized IF protocols to allow comparability between different studies. Furthermore, to identify appropriate interventions tailored for PCOS women, it is important to explore different durations and types of IF (e.g., time-restricted feeding, alternate-day fasting, Ramadan fasting). Additionally, future research should measure a comprehensive set of reproductive (such as AMH, LH, FSH, and testosterone) and metabolic markers (such as insulin resistance and lipid profiles) before and after IF intervention. This can support the role of IF in controlling PCOS. It can also help determine whether PCOS women with different BMI or obesity statuses may exhibit different responses to IF, helping to apply more personalized IF regimens tailored to different PCOS women.

## Figures and Tables

**Table 1:** Summary characteristics of the included studies in this meta-analysis.

Study ID	Study Design	Control Group	Duration of Study (Weeks)	Study Population	Health Status	Age (Years) Range or Mean ± SD	BMI	Type of IF	Duration of IF (Weeks)	Control
Li et al. [13]	Cohort	No	6	Adult females	PCOS and insulin resistant	18–31	Normal and Overweight	16 h	5	Prefasting
Asemi et al. [19]	Quasi (pre-post)	No	4	Mixed adults and adolescents	PCOS	27.5 + 43.6	Overweight and obese	Ramadan fasting	4	Prefasting
Feyzioglu, et al. [18]	cohort	No	6	Adult females	PCOS	25.57 ± 2.67	Obese	8 h	6	Prefasting
Zangeneh et al. [20]	cohort	Yes	4	Adult females	PCOS	Fasting 28.8 ± 3.67 Control 29.4 ± 4.60	Not reported	Ramadan fasting	4	Regular diet

**Table 2:** Summary of the individual results for sex hormones from the included studies in this meta-analysis.

Study	Hormone	Post-Tx (Mean)	Post-Tx (SD)	Pre-Tx (Mean)	Pre-Tx (SD)	Total *n*	*p*-Value
Li et al. [13]	(TT) Total testosterone (ng/mL)	0.87	0.36	1.01	0.36	15	-
	(SHBG) sex hormone-binding globulin (nmol/L)	23.93	13.85	18.60	9.93	15	-
	(FAI) (%) free androgen index	16.2	9.56	21.9	11.17	15	0.001
	LH (mIU/mL)	10.67	5.22	13.09	4.83	15	0.176
	FSH (mIU/mL)	5.01	0.88	5.94	1.50	15	-
Feyzioglu et al. [18]	FSH (mlU/mL)	4.43	1.17	5.22	1.74	30	-
	LH (mlU/mL)	5.29	1.49	9.45	3.70	30	-
	Total testosterone (ng/dL)	0.29	0.21	0.52	0.38	30	-
	SHBG (nmol/L)	73.50	21.70	48.00	24.74	30	-
Zangeneh et al. [20]	FSH (mIU/mL)	5.24	1.72	5.6	1.96	40	-
	LH (mIU/mL)	8.51	5.46	8.08	6.77	40	-
	Testosterone (ng/mL)	1.94	1.97	1.73	1.18	40	-

## Data Availability

All data generated and analyzed during this study are included in this article, and also available from the authors upon reasonable request.

## Supplementary Materials

The following supporting information can be downloaded at: https://www.mdpi.com/article/10.3390/metabo15100654/s1, Results S1: PRISMA 2020 Checklist.

## Appendix A

**Table A1:** Search terms used in the literature review and results retrieved from different databases.

Database	Search Details	Results
PubMed	((“polycystic ovary syndrome”[MeSH Terms] OR (“polycystic”[All Fields] AND “ovary”[All Fields] AND “syndrome”[All Fields]) OR “polycystic ovary syndrome”[All Fields] OR (“polycystic”[All Fields] AND “ovarian”[All Fields] AND “syndrome”[All Fields]) OR “polycystic ovarian syndrome”[All Fields]) AND (“intermittent fasting”[MeSH Terms] OR (“intermittent”[All Fields] AND “fasting”[All Fields]) OR “intermittent fasting”[All Fields] OR (“time”[All Fields] AND “restricted”[All Fields] AND “eating”[All Fields]) OR “time restricted eating”[All Fields])) AND (humans[Filter])	8
	((“polycystic ovary syndrome”[MeSH Terms] OR (“polycystic”[All Fields] AND “ovary”[All Fields] AND “syndrome”[All Fields]) OR “polycystic ovary syndrome”[All Fields] OR (“polycystic”[All Fields] AND “ovarian”[All Fields] AND “syndrome”[All Fields]) OR “polycystic ovarian syndrome”[All Fields]) AND “ramadan”[All Fields]) AND (humans[Filter])	5
	((“polycystic ovary syndrome”[MeSH Terms] OR (“polycystic”[All Fields] AND “ovary”[All Fields] AND “syndrome”[All Fields]) OR “polycystic ovary syndrome”[All Fields] OR (“polycystic”[All Fields] AND “ovarian”[All Fields] AND “syndrome”[All Fields]) OR “polycystic ovarian syndrome”[All Fields]) AND (“fasted”[All Fields] OR “fasting”[MeSH Terms] OR “fasting”[All Fields] OR “fastings”[All Fields] OR “fasts”[All Fields])) AND (humans[Filter])	1998
	(“polycystic ovary syndrome”[MeSH Terms] OR (“polycystic”[All Fields] AND “ovary”[All Fields] AND “syndrome”[All Fields]) OR “polycystic ovary syndrome”[All Fields] OR (“polycystic”[All Fields] AND “ovarian”[All Fields] AND “syndrome”[All Fields]) OR “polycystic ovarian syndrome”[All Fields]) AND ((“meals”[MeSH Terms] OR “meals”[All Fields] OR “meal”[All Fields]) AND (“epidemiology”[MeSH Subheading] OR “epidemiology”[All Fields] OR “frequency”[All Fields] OR “epidemiology”[MeSH Terms] OR “frequence”[All Fields] OR “frequences”[All Fields] OR “frequencies”[All Fields]))	13
	(“polycystic ovary syndrome”[MeSH Terms] OR (“polycystic”[All Fields] AND “ovary”[All Fields] AND “syndrome”[All Fields]) OR “polycystic ovary syndrome”[All Fields] OR (“polycystic”[All Fields] AND “ovarian”[All Fields] AND “syndrome”[All Fields]) OR “polycystic ovarian syndrome”[All Fields]) AND (“intermittent fasting”[MeSH Terms] OR (“intermittent”[All Fields] AND “fasting”[All Fields]) OR “intermittent fasting”[All Fields] OR (“time”[All Fields] AND “restricted”[All Fields] AND “feeding”[All Fields]) OR “time restricted feeding”[All Fields])	14
Scopus	(“polycystic ovary syndrome” AND (“intermittent fasting” OR “time restricted eating” OR “time restricted feeding “OR” meal frequency”))	15
	(polycystic ovarian syndrome) AND (fasting)	2693
Web of Science	polycystic ovary syndrome AND fasting	2500
	(“polycystic ovary syndrome” AND (“intermittent fasting” OR “time restricted eating” OR “time restricted feeding”))	8
	polycystic ovary syndrome AND Ramadan	1
	polycystic ovary syndrome AND meal frequency	5
